# Supplementary material for: HN1 Is Enriched in the S-Phase, Phosphorylated in Mitosis, and Contributes to Cyclin B1 Degradation in Prostate Cancer Cells
Source: Biology (Basel). 2023 Jan 26;12(2):189. doi: 10.3390/biology12020189 (PMC9952942; doi:10.3390/biology12020189)
Supplement: Supplementary file 1 [file biology-12-00189-s001.zip › biology-2141533-Figure S7.pdf]

(a)

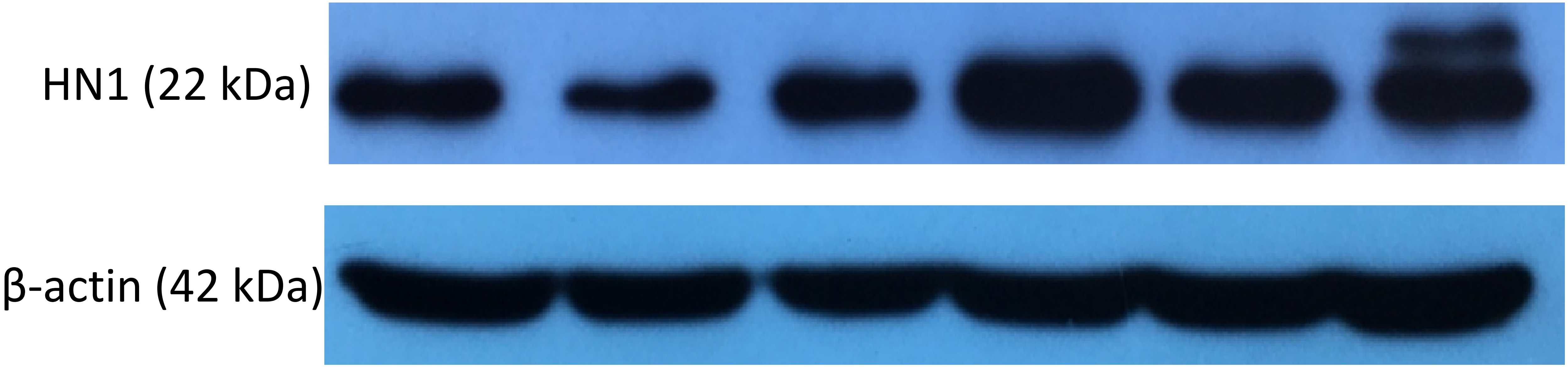

(b)

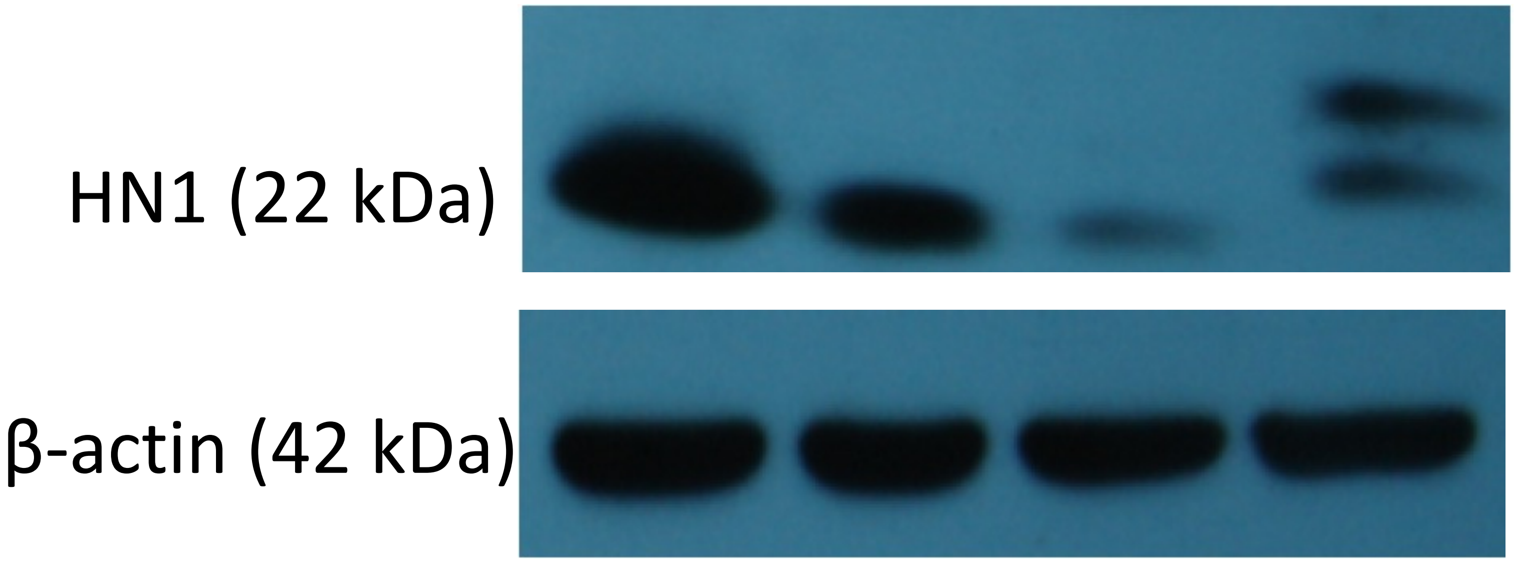

(c)

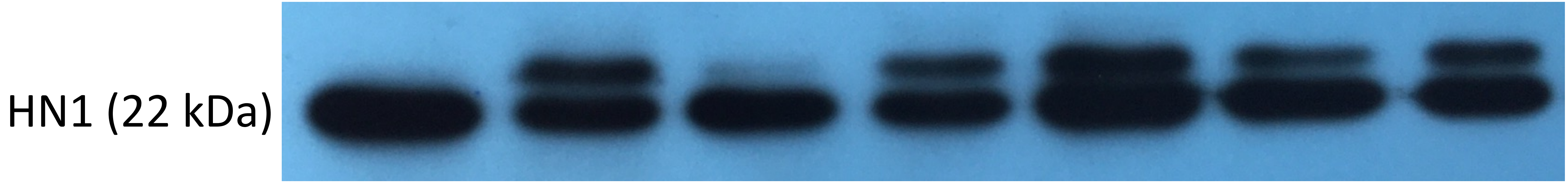

(d)

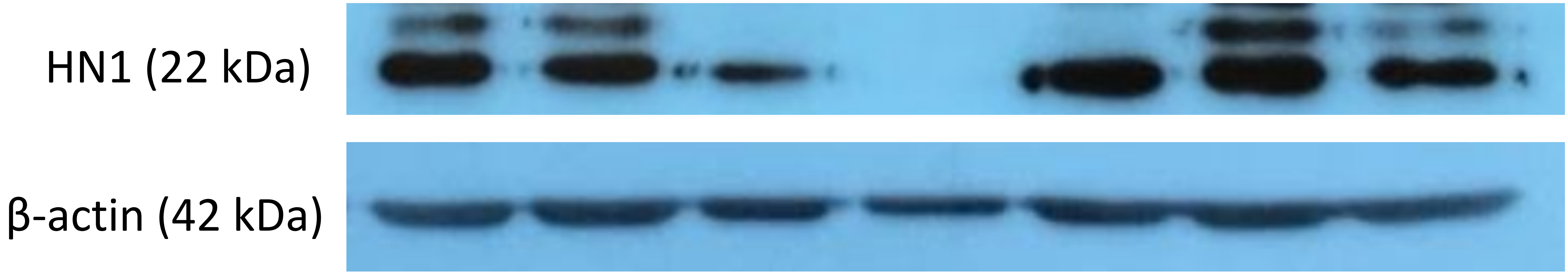

(e)

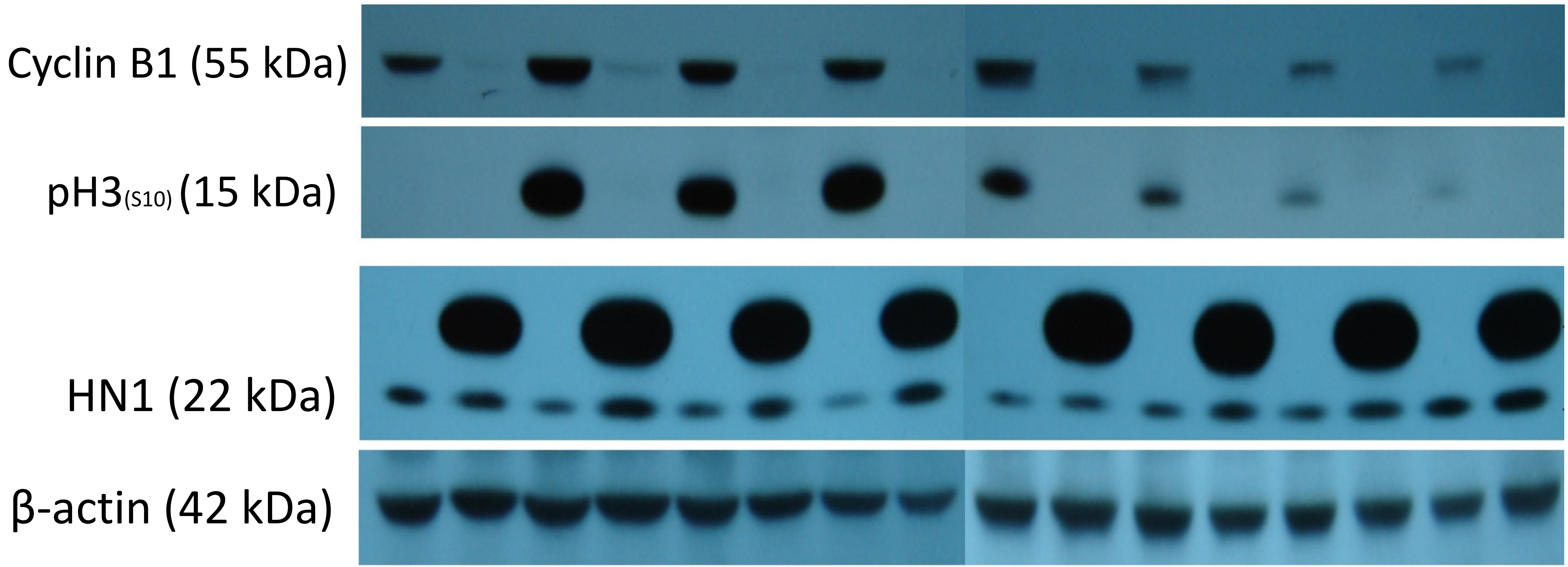

(f)

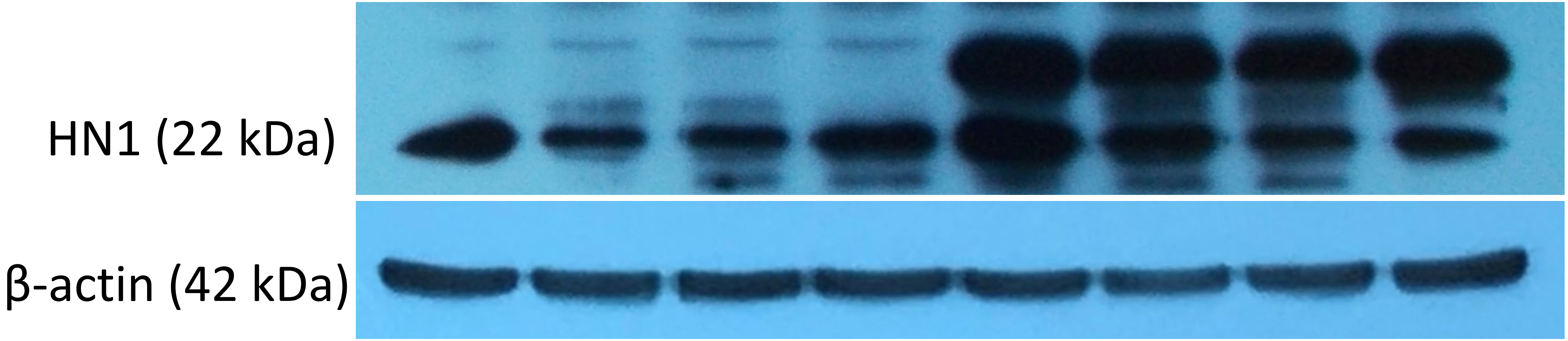

(g)

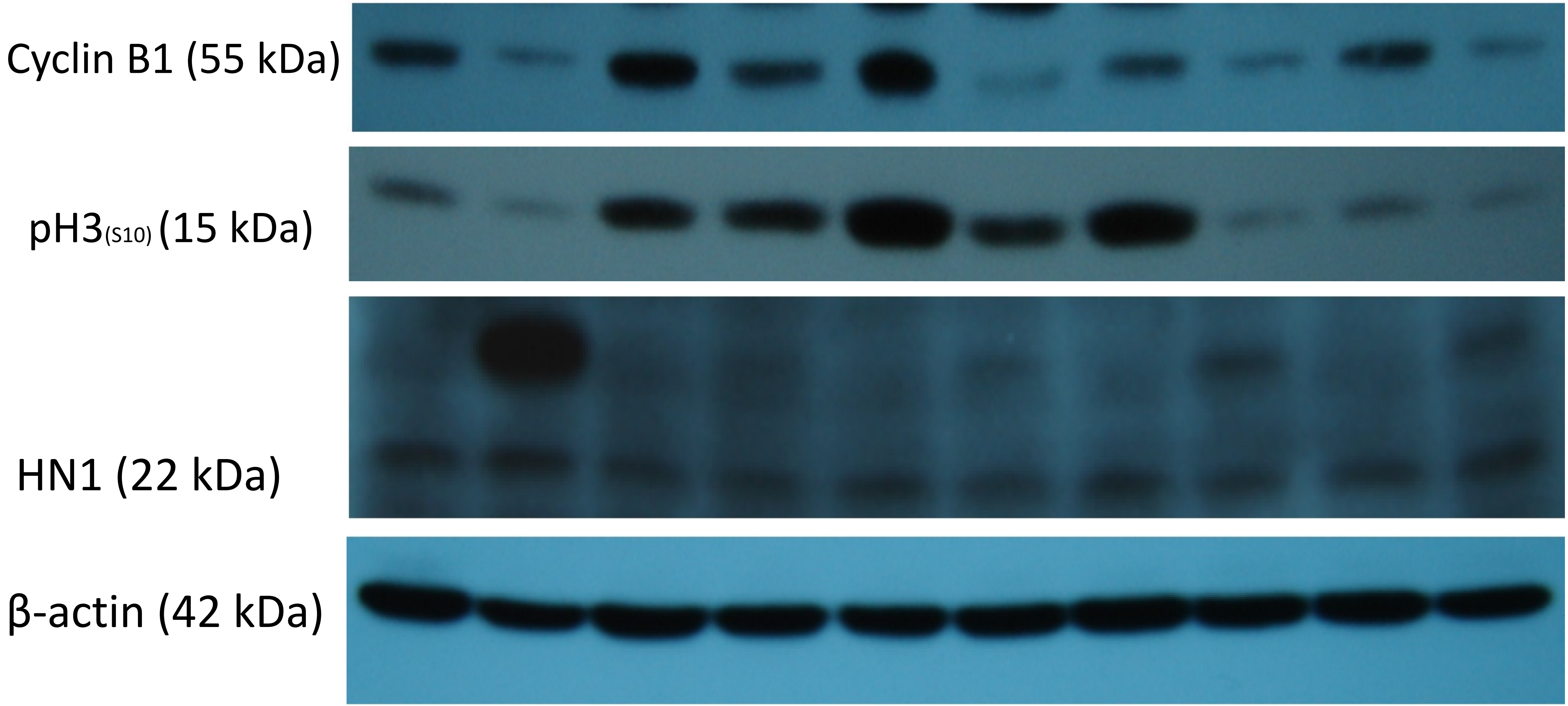

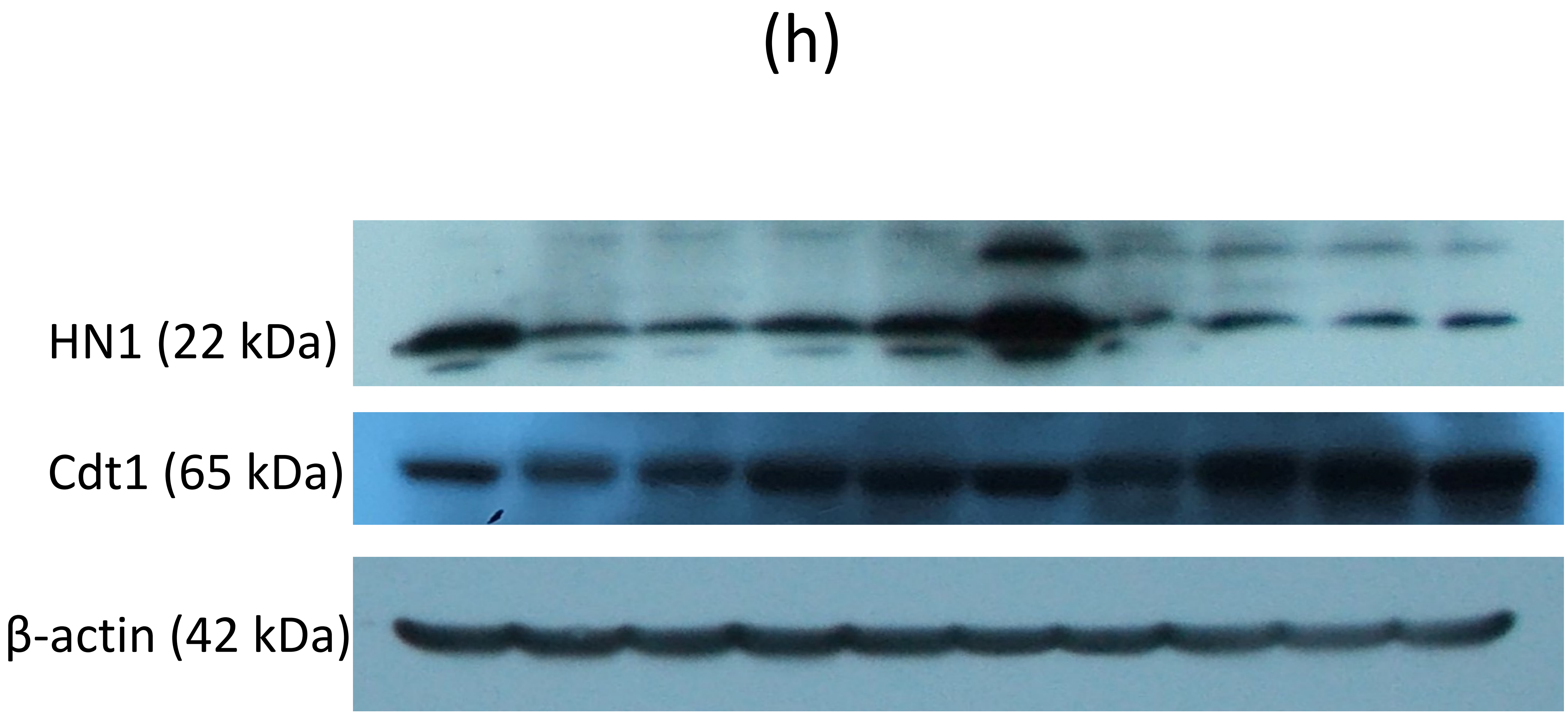
(h)
HN1 (22 kDa)
Cdt1 (65 kDa)
β-actin (42 kDa)

(i)

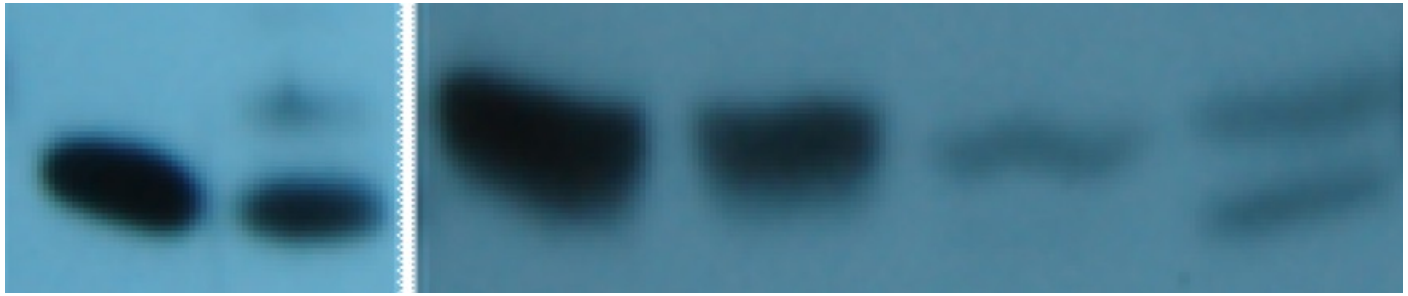

HN1 (22 kDa)

(j)

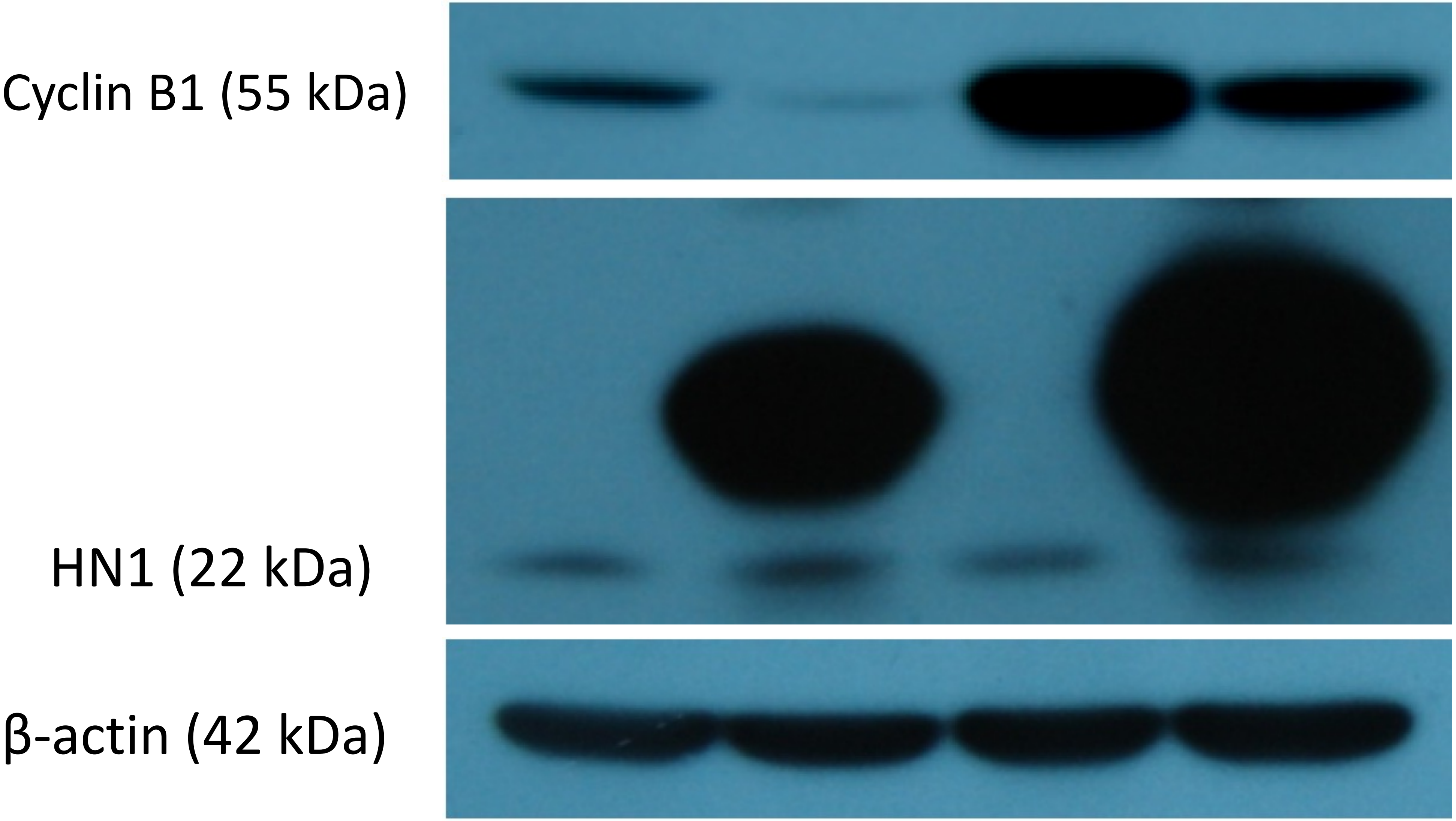

(k)

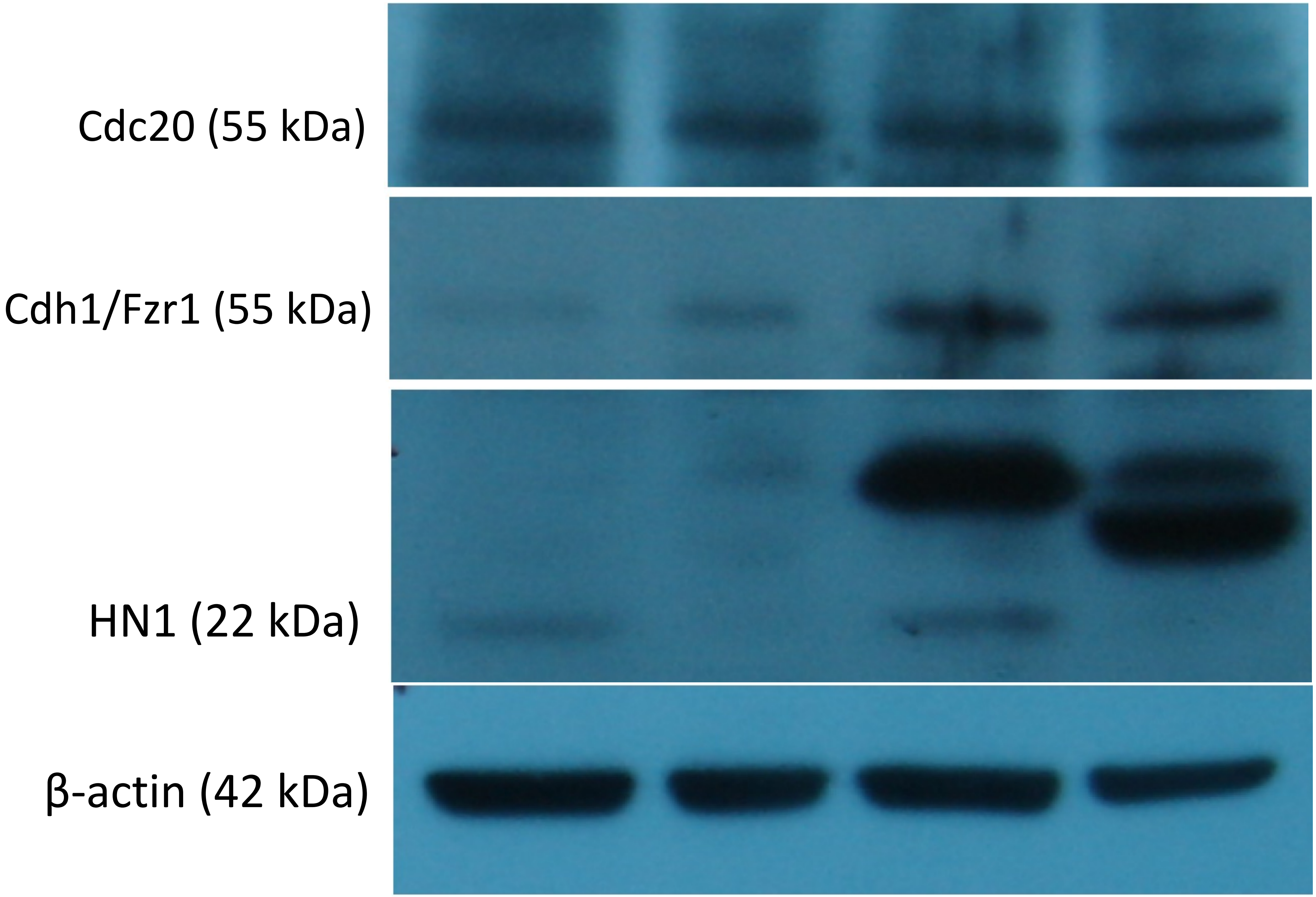

(I)

55 kDa

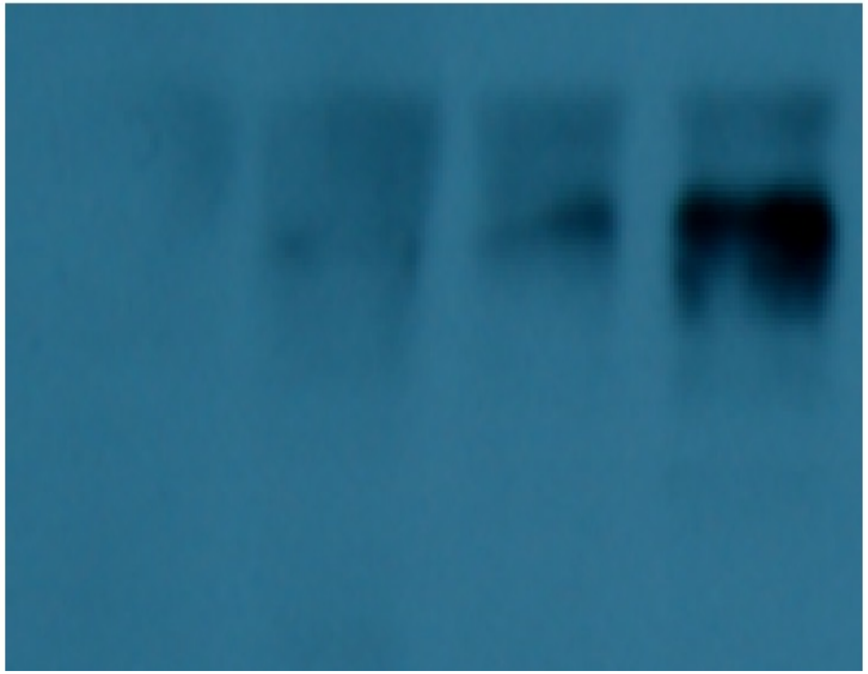

Ubiquitin’ Cyclin B1 (>55 kDa)

(m)

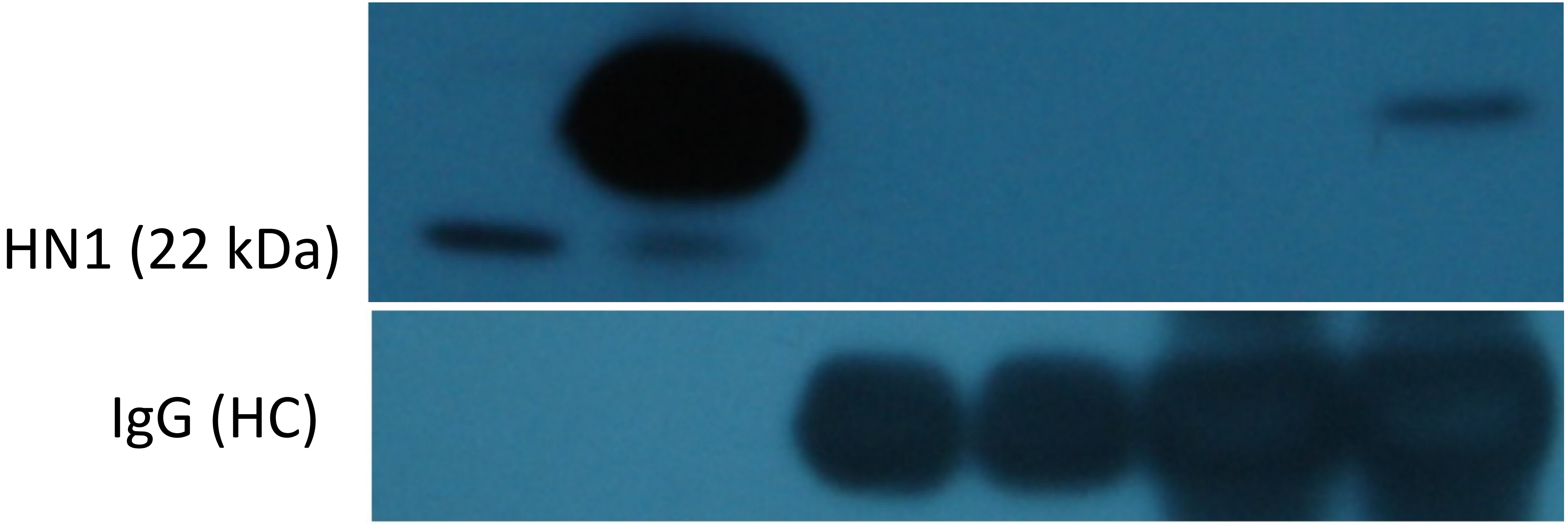

(n)

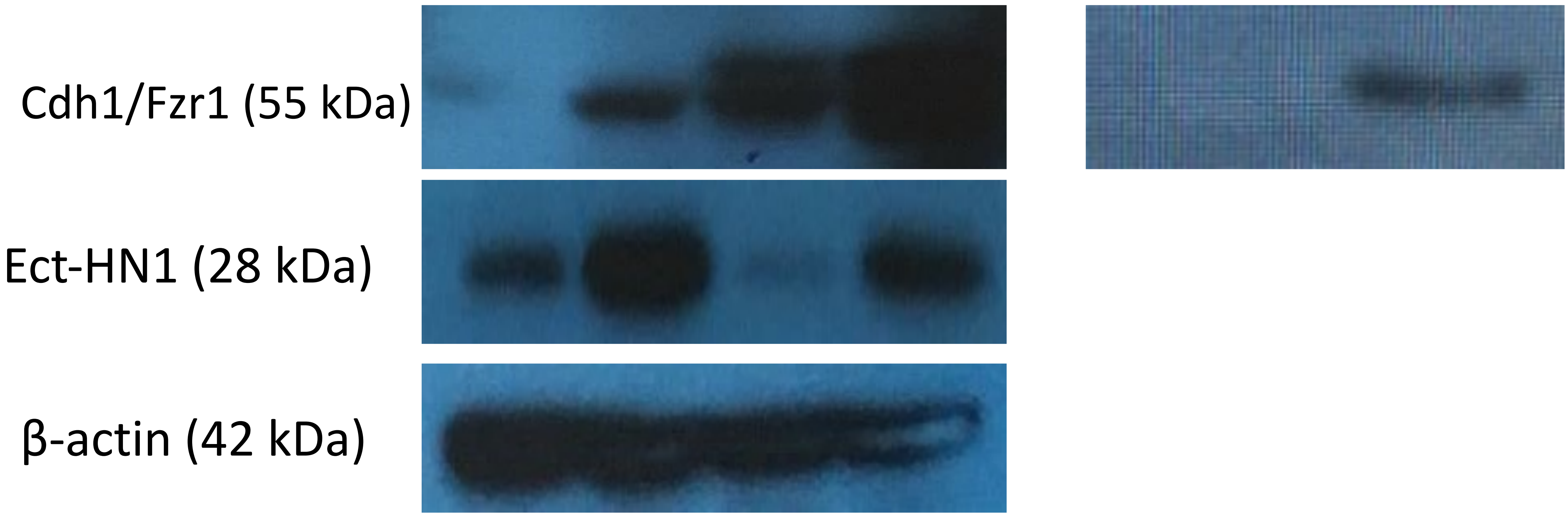

(o)

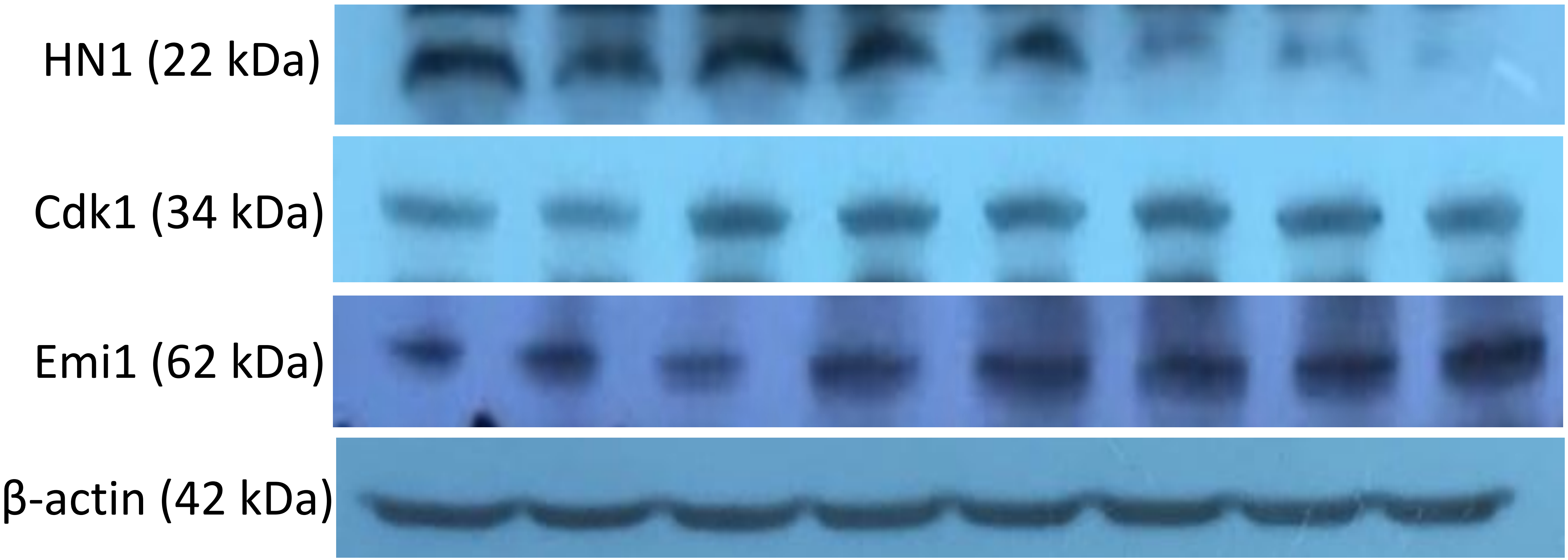

(p)

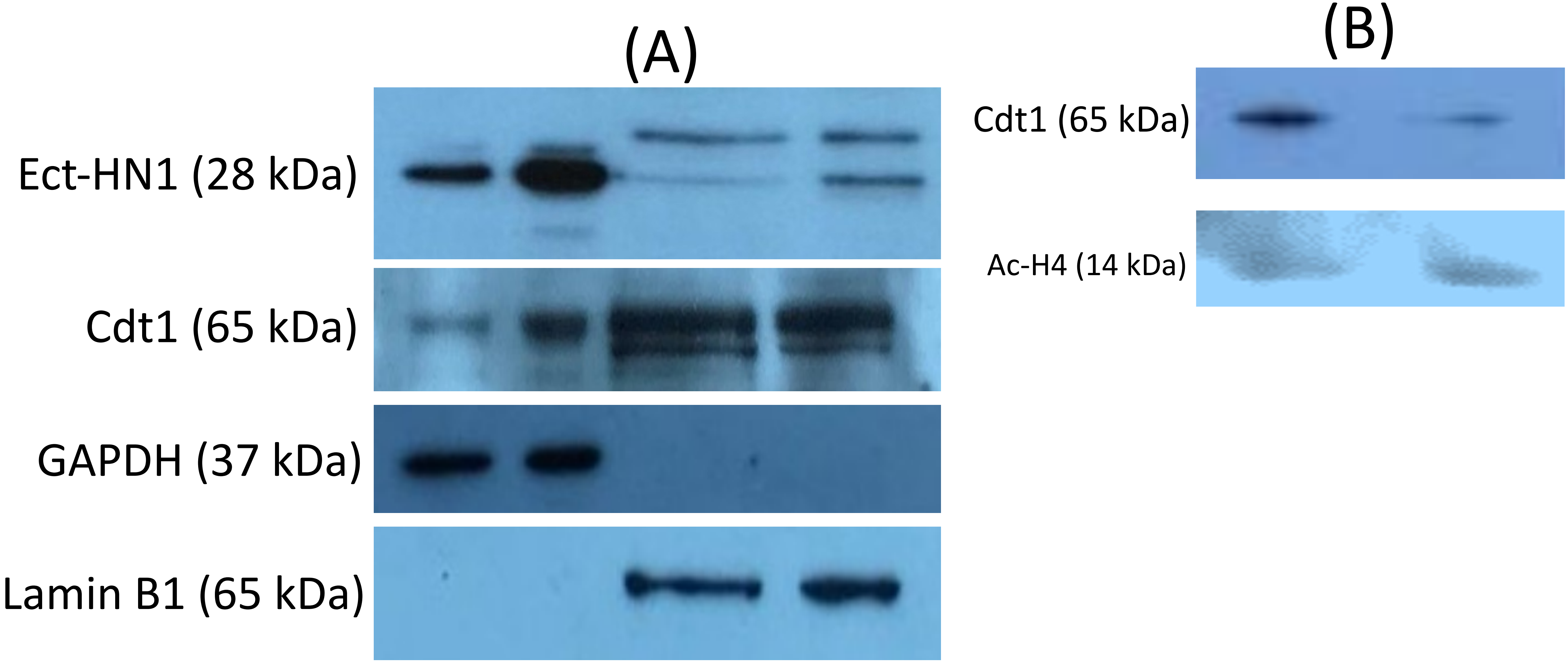
(A)
Ect-HN1 (28 kDa)
Cdt1 (65 kDa)
GAPDH (37 kDa)
Lamin B1 (65 kDa)
(B)
Cdt1 (65 kDa)
Ac-H4 (14 kDa)

Figure S7: Original images of western blots. (a): the original image of western blots for figure 1A; (b): figure 2A; (c): figure 2B; (d): figure 2C; (e): figure 3A; (f): figure 3C; (g): figure 4A; (h): figure 4C; (i): figure 5A; (j): figure 5B; (k): figure 6A; (l): figure 5C; (m): figure 6B; (n): figure 6C; (o): figure 7; (p): figure 9A and figure 9B.
