# Supplementary material for: HN1 Is Enriched in the S-Phase, Phosphorylated in Mitosis, and Contributes to Cyclin B1 Degradation in Prostate Cancer Cells
Source: Biology (Basel). 2023 Jan 26;12(2):189. doi: 10.3390/biology12020189 (PMC9952942; doi:10.3390/biology12020189)
Supplement: Supplementary file 1 [file biology-12-00189-s001.zip › biology-2141533-supplementary.pdf]

Supplementary Material

HN1 is Enriched in S-phase, Phosphorylated in Mitosis, and Contributes to Cyclin B1 Degradation in Prostate Cancer Cells

Aadil Javed[a], Gülseren Özduman[a], Lokman Varisli[a], Bilge Esin Özturk[a], Kemal Sami Korkmaz[a#]

[a]Ege University, Faculty of Engineering, Department of Bioengineering, Cancer Biology Laboratory, Bornova, Izmir, Turkey

[#]Address correspondence to Prof. Dr. Kemal Sami Korkmaz, ks_korkmaz@yahoo.com; kemal.sami.korkmaz@ege.edu.tr

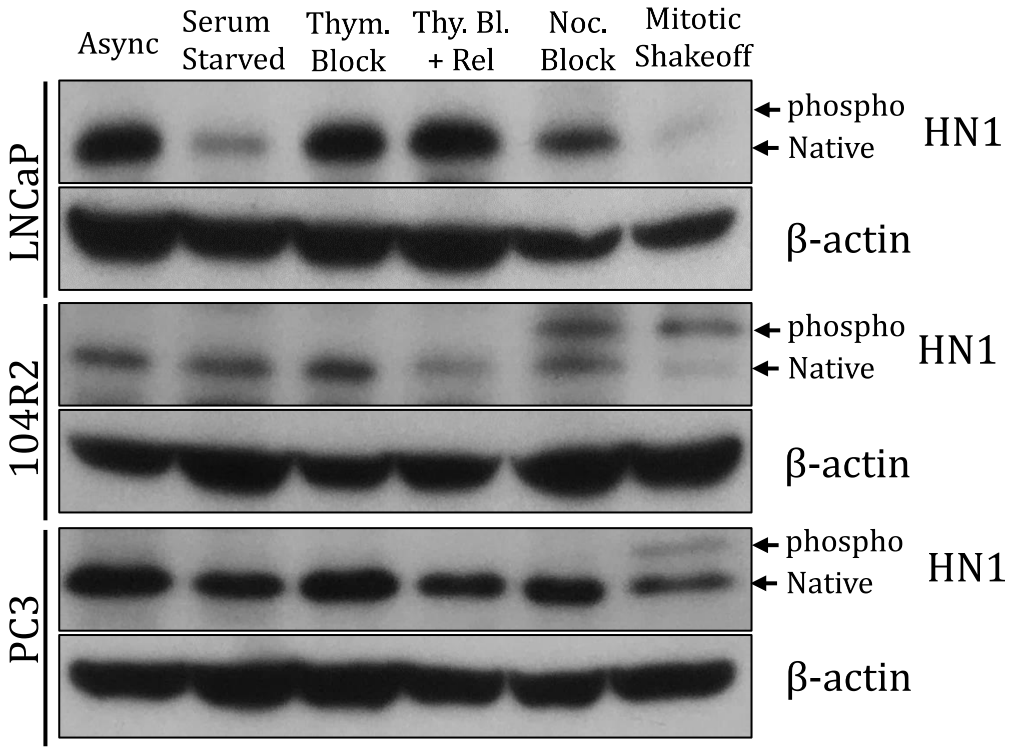

Figure S1: HN1 protein levels in different phases of cell cycle in LNCaP, LNCaP-104R2, and PC3 cell lines. Cells were synchronized in different phases of the cell cycle by serum starvation, Thymidine block, Thymidine block, and release for 2 hours in fresh media, Nocodazole block, and mitotic shake-off approaches. Protein lysates collected from the aforementioned treatments were analyzed by western blotting by staining with anti-HN1 and anti-β-actin antibodies. HN1 levels were enriched in the early S phase as observed in the Thymidine block sample and the Thymidine block and release sample as compared to other phases. In mitosis, the distinct band of HN1 labeled as phospho-HN1 was also observed.

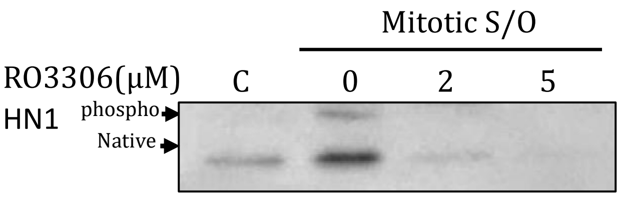

Figure S2: Phospho-HN1 disappears upon RO-3306 (CDKi) treatment. Mitotic populations of PC3 cells were further treated with lower concentrations of RO-3306 (CDKi) (0, 2, and 5 µM) and protein lysates were subjected to western blotting whereupon HN1 levels were measured.

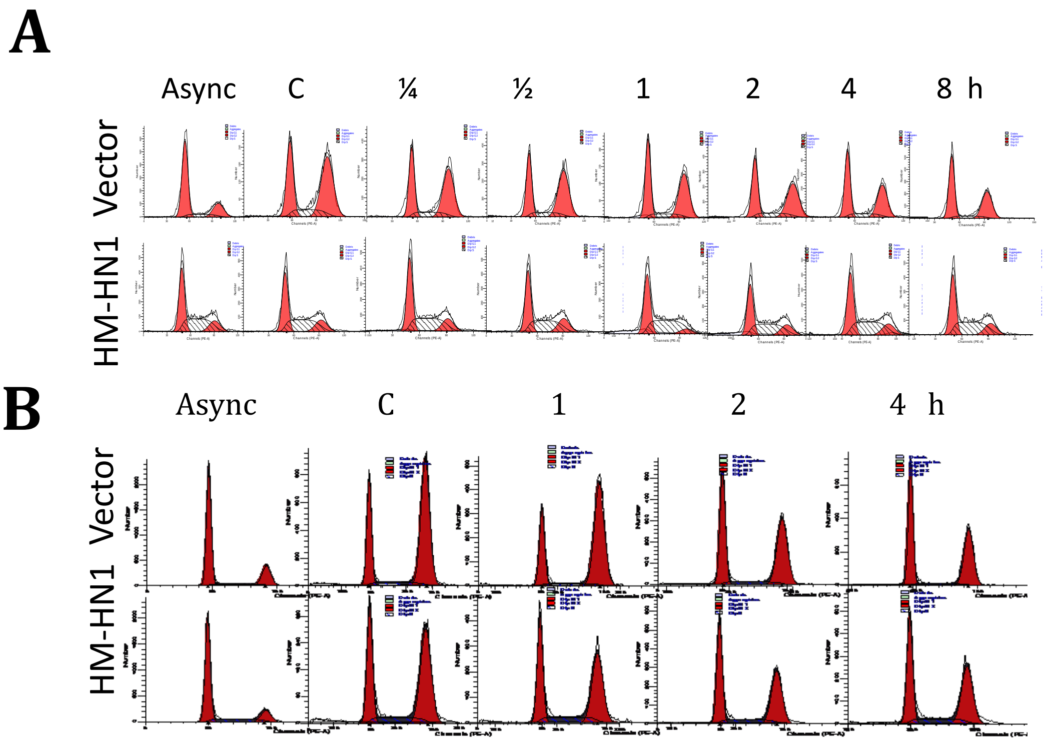

Figure S3: HN1 overexpression using plasmid DNA before and after G2 phases using Nocodazole synchronization. (A) After 16 h of Nocodazole treatment, cells were released from the Nocodazole block and were analyzed using flow cytometry 0, 15, and 30 min and at 1, 4, and 8 h later. When HN1 was ectopically expressed before the Nocodazole block, a considerable number of cells accumulated in the S phase. (B) Nocodazole-treated PC3 cells were released from Nocodazole block 0, 1, 2, or 4 h after a 24-h transfection with the control or pcDNA4-HM-HN1 construct and were analyzed using flow cytometry. The cell cycle phases were identified using propidium iodide (PI) staining. The HN1 overexpression after G2 (and within Nocodazole block), led to the appearance of G1 faster in HN1 overexpressed cells as compared to vector-transfected cells.

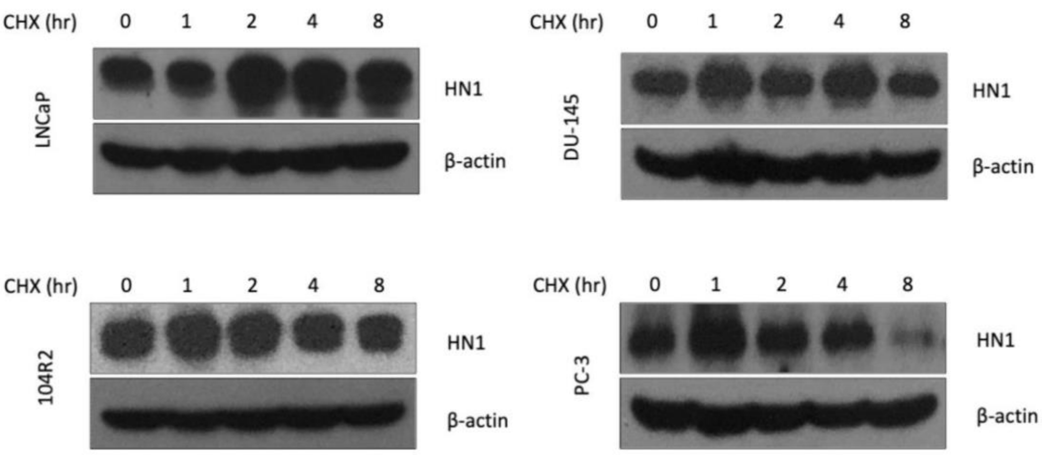

Figure S4: Cyclohexamide (CHX) chase assay for HN1 in different Prostate cancer cell lines. HN1 stays in a stabilized form for longer in LNCaP, 104R2, and DU-145 cells as compared to PC3 cells. β-actin was stained as a control for western blot. 4 X 10^5 cells from each cell line were seeded on 6 cm^2 cell culture dishes and grown for 2 days. 100 µg/ml cycloheximide (CHX) dissolved in ethanol was used to inhibit the transcriptional machinery of the cells, and to determine the impact on native HN1 protein levels. Cells were treated for up to 8 hours with cycloheximide and harvested by cell scraping as described previously. After isolating proteins, total protein lysate (µg) was loaded on a 12 % SDS-Gel for the separation of proteins. After electrophoresis, the proteins were transferred to a PVDF membrane for subsequent immunoblotting. The anti-HN1 antibody and anti-β-actin antibodies were used. HN1 levels remained high in LNCaP cells even after 8 hours of cycloheximide treatment. In LNCaP-104R2 cells, HN1 levels initiate to go down after 4 hours and remained low at 8 hours of CHX exposure. In DU-145 cells, HN1 protein levels varied among different hours, however eventually went down in 8 hours of CHX treatment. Interestingly, HN1 native protein levels significantly reduce from 1 to 2 hours and almost completely disappear at 8 hour time period of CHX exposure. This data indicates that HN1 is a relatively short-lived protein in PC3 cells as compared to other Prostate cancer cell lines.

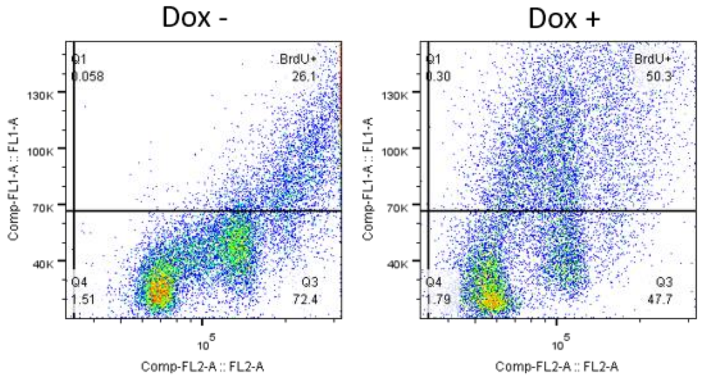

Figure S5: The flow cytometry analysis of PC3-pCW57-HM-HN1 cells. The cells were treated with doxycycline for 2 days and collected using trypsinization and fixed with ethanol. Anti-BrdU and PI staining were performed and cells were gated in Flowjo v10 for analyzing BrdU+ cells. This is the representative figure of the analysis.

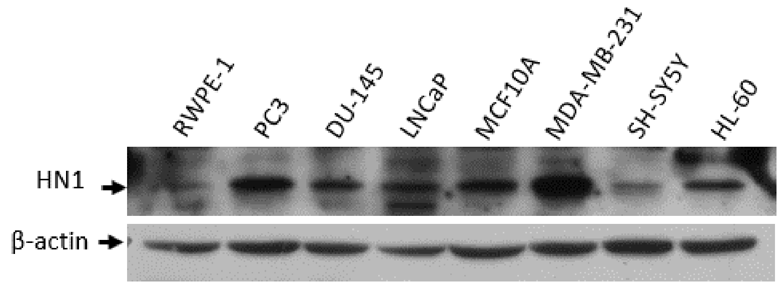

Figure S6: HN1 relative protein levels in different cell lines: 50 μg of protein lysate from different cell lines were subjected to western blotting using anti-HN1 and anti-β-actin antibodies. HN1 was observed as highly expressed in Prostate cancer cell lines PC3, DU-145, and LNCaP as compared to normal Prostate epithelial cells RWPE-1. Moreover, HN1 levels in aggressive Breast Cancer cell line MDA-MB-231 were considerably higher as compared to normal epithelial Breast cells MCF10A. Other cell lines checked include SHSY-5Y and HL-60.
